# Thrombosis of the Abdominal Veins in Childhood

**DOI:** 10.3389/fped.2017.00188

**Published:** 2017-09-05

**Authors:** Riten Kumar, Bryce A. Kerlin

**Affiliations:** ^1^Department of Pediatrics, The Ohio State University College of Medicine, Columbus, OH, United States; ^2^Division of Pediatric Hematology/Oncology/BMT, Nationwide Children’s Hospital, Columbus, OH, United States; ^3^Center for Clinical and Translational Research, The Research Institute at Nationwide Children’s Hospital, Columbus, OH, United States

**Keywords:** thrombosis, inferior vena cava, portal vein, mesenteric vein, Budd–Chiari, renal vein

## Abstract

Abdominal venous thrombosis is a rare form of venous thromboembolic disease in children. While mortality rates are low, a significant proportion of affected children may suffer long-term morbidity. Additionally, given the infrequency of these thrombi, there is lack of stringent research data and evidence-based treatment guidelines. Nonetheless, pediatric hematologists and other subspecialists are likely to encounter these problems in practice. This review is therefore intended to provide a useful guide on the clinical diagnosis and management of children with these rare forms of venous thromboembolic disease. Herein, we will thus appraise the current knowledge regarding major forms of abdominal venous thrombosis in children. The discussion will focus on the epidemiology, presentation, diagnosis, management, and outcomes of (1) inferior vena cava, (2) portal, (3) mesenteric, (4) hepatic, and (5) renal vein thrombosis.

## Introduction

Though rare in children, abdominal venous thromboembolism (VTE) may result in substantial morbidity. In our recent epidemiologic study, there were 4,538 pediatric VTE discharges in 1 year (1). Of these, 17% were abdominal VTE, 44% involved the inferior vena cava (IVC), 36% portal veins, 11% hepatic veins, and 10% were renal vein thrombi. In a prospective study of 466 childhood VTE, 12% were abdominal; 46% of which were in the IVC (2). Thus, 12–17% of childhood VTE is intra-abdominal with IVC thrombosis predominating. High-quality data are limited, thus the following recommendations are based on available literature, expert consensus, and personal experience. The use of anticoagulants in pediatric VTE is comprehensively reviewed in another manuscript in this collection (3).

## IVC Thrombosis

The majority of IVC thrombi result from extension of ilio-femoral deep vein thrombi (DVT) which, in turn, are a common complication of femoral vein catheterization (4–7). Neonates are prone to catheter-related IVC thrombosis, due to the common utilization of lower extremity and umbilical venous catheters (UVCs). In a Dutch study, 66% of 44 catheter-related thrombi in neonates involved the IVC or right atrium whereas the IVC was not involved in non-catheter-related neonatal VTE (5). In a Canadian registry of catheter-related VTE in children of all ages, IVC involvement was appreciated in 10% of the cohort (6). In the PROTEKT trial, 29% of catheter-related VTE were found in the lower extremity venous system, and one-third of these extended into either the superior or inferior vena cava (7). Thus, it is important to identify the proximal extension of all catheter-related VTE to define the anatomic scope of involvement.

Congenital IVC agenesis/atresia, which has an estimated prevalence of ~0.6%, may present with calcified IVC thrombosis during infancy (8–11). This is most commonly an incidental imaging finding in an asymptomatic infant. However, IVC anomalies occasionally present during adolescence as what initially appears to be unprovoked, proximal lower extremity DVT (12–14). Two recent studies which included adolescents, found that 11–13% of IVC thromboses had evidence of congenital IVC anomaly, whereas none of the control group (cases of lower extremity DVT without IVC involvement) had anomalies (15, 16). In cases of delayed presentation, it is thought that collateral venous drainage is accomplished *via* deep, median, portal, or superficial abdominal veins (17). It is reasonable to hypothesize an anatomic thrombophilia due to increased vascular resistance in these smaller veins, which predisposes to lower extremity DVT. Therefore, it is appropriate to maintain a high index of suspicion for IVC anomalies in children with lower extremity DVT who have no apparent thrombotic predisposition. IVC thrombosis has also been reported following physical abuse and major abdominal surgery in children (18–21).

Doppler ultrasonography, the imaging modality of choice for extremity thrombosis, may miss isolated pathology of the IVC; thus computed tomography (CT) and magnetic resonance scans are preferable (22). Anticoagulant therapy for a period of 3–6 months is appropriate for provoked and unprovoked initial episodes (23). Some authors suggest an initial course of 12 months given concerns for significant post-thrombotic syndrome (PTS) with persistent IVC thrombosis (24, 25). Additionally, some experts advocate thrombolytic therapy as initial treatment (26, 27). It is thought that such an aggressive intervention may decrease the risk for PTS, pulmonary embolism, or acute renal failure (in the setting of suprarenal IVC thrombosis). However, this strategy is based upon expert consensus and thus awaits clinical trial data. Successful IVC stenting followed by aspirin prophylaxis has been reported in children with congenital heart disease and subacute/chronic thrombotic IVC obstruction (28). With regard to IVC agenesis/atresia, IVC reconstruction utilizing polytetrafluoroethylene grafts has demonstrated 83% patency with stable or improved PTS scores at 41 months (29).

**Figure 1:**
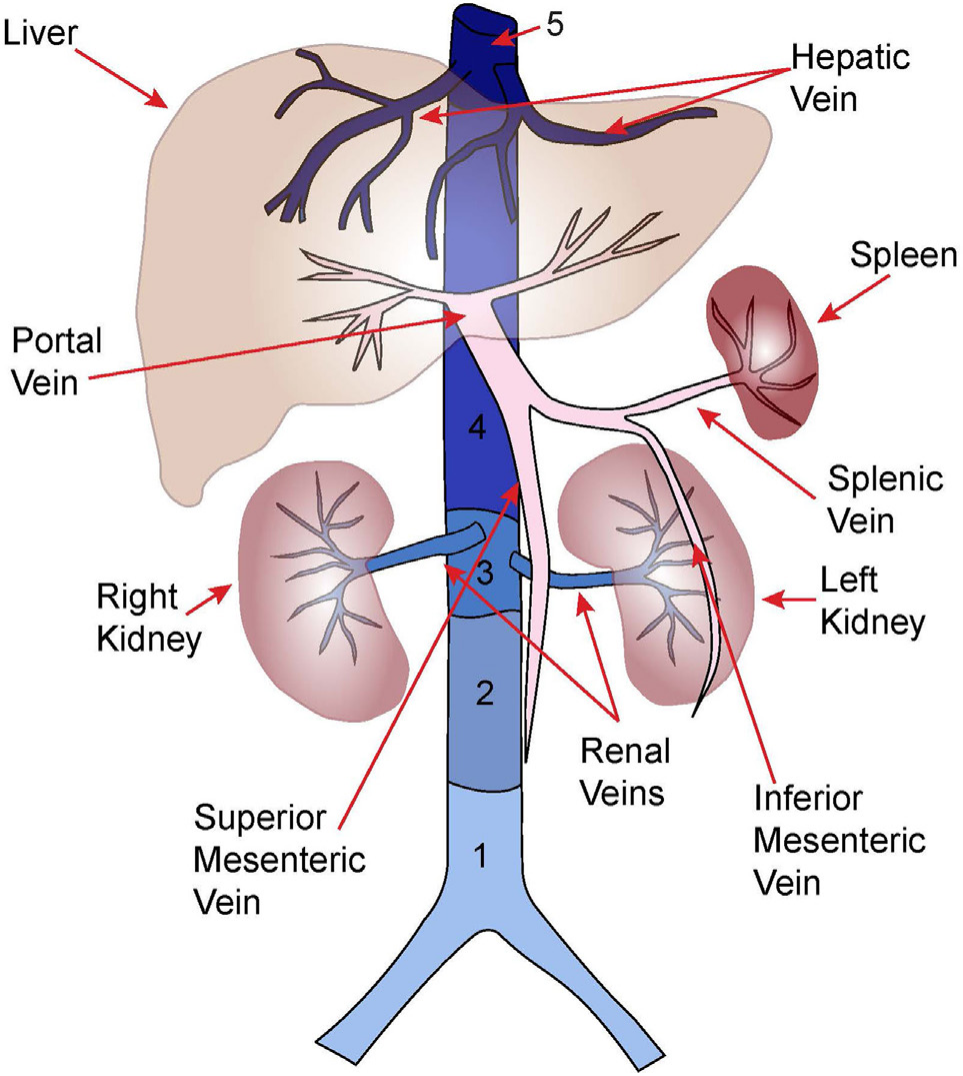
Anatomy of major abdominal veins. Inferior vena cava segments adapted from Ref. (8).

Long-term IVC thrombosis outcome has been studied in 39 children who were followed for a maximum of 18 years (24). Six patients (15%) died within 3 months of diagnosis. Twenty-one (53%) presented with extensive thrombosis (defined as involving ≥2 IVC segments or extending into the iliac veins; Figure 1). Complete reconstitution of IVC flow was observed in only four (19%) children; three of whom had undergone intervention [thrombolysis (1); surgical (2)]. Persistent caval and/or iliac obstruction was observed in the remaining 17 (81%) patients (6 of whom had been treated with thrombolysis). The remaining 12 (30%) had complete resolution of limited IVC thrombosis on follow-up. PTS was frequent (30%) in those with persistent IVC pathology (24).

## Portal Vein Thrombosis (PVT)

Portal vein thrombosis refers to partial or complete occlusion of the portal venous system (Figure 1). Thrombus may be restricted to the portal veins or may extend into the splenic and/or superior mesenteric veins. PVT often occurs during the neonatal period with an estimated incidence of 1.3/100,000 live births, and 36/1,000 NICU admissions (9, 30). However, neonatal PVT is often asymptomatic and only identified in the subset of patients who develop symptomatic portal hypertension [e.g., gastrointestinal (GI) bleeding, splenomegaly] several years after the initial thrombotic event (31, 32). Thus, the true incidence is likely higher.

In neonates, UVC placement is the most common cause for PVT. In a retrospective Canadian study of 133 neonates with PVT, 73% were UVC-associated (30). In contrast, incidence of UVC-associated PVT in prospective studies ranges from 0 to 43% (33–37). This variation may be explained by study design differences, diagnostic criteria, and timing of imaging. Other risk factors for neonatal PVT include prolonged catheterization, position of catheter tip, transfusion through the UVC, and sepsis (30, 35, 36). In older children and adults, etiologies of PVT include pancreatitis, cirrhosis, liver transplant, splenectomy, and sickle cell disease (32). Notably, ~50% of childhood PVT has no identifiable etiology (31, 32).

Because neonatal PVT is often asymptomatic, the diagnosis is often an incidental finding of imaging performed for other indications. In the Canadian study, the most common indications for imaging were thrombocytopenia (20%), abdominal distension (17%), elevated liver enzymes (7%), and hepatosplenomegaly (4%) (30). Many cases of neonatal PVT are diagnosed later in childhood secondary to symptoms of portal hypertension. It is estimated that 5–20% of childhood portal hypertension cases are secondary to PVT (38). In a retrospective study of 108 children with chronic PVT, 52% presented with splenomegaly and 46% with GI hemorrhage (31). Eventually, 79% of the children developed GI bleeding.

Doppler ultrasonography is the most common imaging modality used to diagnose PVT. Acute PVT may be graded ultrasonographically: (a) Grade I: non-occlusive single-branch PVT with normal liver parenchyma, (b) Grade II: occlusive single-branch PVT with normal liver parenchyma, and (c) Grade III: occlusive PVT involving two-branches or occlusive single-branch PVT with liver parenchymal changes (30). An association between Grade III PVT and poor outcome (portal hypertension or liver lobe atrophy) was noted in the original study (30), though a follow-up study failed to confirm this finding (39). Pancreatico-duodenal and peribilliary collaterals may develop around the obstructed veins and are known as “cavernous transformation.” This process begins within 5 weeks after the thrombotic event but may take months-to-years to become apparent ultrasonographically (40). Procoagulant defects have been reported in a substantial proportion of patients with PVT (Table 1) (9, 39, 41–43). In adolescents with unprovoked PVT, we suggest testing for paroxysmal nocturnal hemoglobinuria and Janus tyrosine-kinase 2 (JAK2) mutations (44, 45).

**Table 1:** Studies investigating the association between thrombophilia and abdominal vein thrombosis.

Reference	Year of publication	Type of study	Subjects tested	Thrombophilia identified (%)	OR (95% CI)
**Portal vein thrombosis**					
Heller et al. (9)	2000	Case-control	24	FV Leiden: 4 (17%), PC deficiency: 1 (4%), AT deficiency: 1 (4%)	5.47 (1.7–17.6)
El-Karaksy et al. (41)	2003	Case-control	40	FV Leiden: 12 (30%), PC deficiency: 11 (28%), PG20210: 6 (15%), AT deficiency: 1 (3%)	6 (for FV Leiden only)
Morag et al. (39)	2011	Cohort	25	PS deficiency: 2 (8%), FV Leiden: 2 (8%), AT deficiency: 1 (4%)	NA
Pietrobattista et al. (43)	2010	Case-control	31	PC deficiency: 4 (13%), PS deficiency: 4 (13%), PG20210: 3 (10%), FV Leiden: 2 (6%)	11.91 (1.4–100.7)
Ferri et al. (32, 42)	2012	Cohort	32	APLA: 2 (6%), FV Leiden + PG20210:1 (3%)	NA
**Neonatal renal vein thrombosis**					
Heller et al. (9)	2000	Case-control	31	FV Leiden: 9 (29%), PC deficiency: 2 (7%), AT deficiency: 1 (3%)	10.9 (3.9–31.1)
Kuhle et al. (46)	2004	Cross-sectional	21	FV Leiden: 8 (38%), PG20210 (homozygous): 1 (5%)	NA
Kosch et al. (47)	2004	Case-control	59	FV Leiden: 22 (38%), PG20210: 5 (9%), PC deficiency: 3 (5%), AT deficiency: 3 (5%)	15.6 (7.2–34.2)
Marks et al. (48)	2005	Cohort	28	PC/PS deficiency: 5 (18%), FV Leiden: 5 (18%), PG20210: 1 (4%), AT deficiency: 1 (4%)	NA
Winyard et al. (49)	2006	Cohort	18	FV Leiden: 4 (22%), PC + PS deficiency: 1 (6%)	NA
**Hepatic vein thrombosis**					
Heller et al. (9)	2000	Case-control	10	FV Leiden: 1 (10%)	3.3 (0.6–18.7)
Nagral et al. (50)	2010	Cohort	16	PC deficiency: 2 (13%)^a^, AT deficiency: 1 (6%), APLA: 1 (6%)	NA
Kathuria et al. (51)	2014	Cohort	12	PC deficiency: 6 (50%)^a^, PS deficiency: 3 (25%)^a^, APLA syndrome: 3 (25%)^b^, hyperhomocysteinemia: 2 (17%), AT deficiency: 1 (8%)^a^	NA

*PC, protein C; PS, protein S; AT, antithrombin; APLA, anti-phospholipid antibody syndrome; FV Leiden, Factor V Leiden mutation (*FV R506Q*); PG20210, prothrombin gene mutation (*FII G20210A*); NA, not applicable*.

*^a^Low PC, PS, and AT levels may be acquired in the setting of advanced liver disease*.

*^b^Unclear if abnormal labs were repeated 12 weeks apart to confirm APLA syndrome. Note: pediatric data regarding the association of thrombophilia with IVC thrombosis/atresia and mesenteric vein thrombosis not available*.

The utility of anticoagulation for PVT remains unclear since a significant proportion resolve spontaneously. In the Canadian study, 77% of non-occlusive PVT resolved completely or partially whereas only 48% of occlusive PVT resolved. Anticoagulation was administered to 44% of the cohort, but did not appear to influence outcome (30). Similarly, a 70% resolution rate was appreciated for non-occlusive PVT vs. only 31% for occlusive PVT in another study (35). Given the ambiguity surrounding the role of anticoagulation and high rate of spontaneous resolution, there are no consensus guidelines for PVT management (23). Thus, anticoagulation should be considered on a case-by-case basis. In neonates with acute, occlusive PVT and no contraindications, 6–12 weeks of anticoagulation is reasonable. Conversely, in patients who are at risk for bleeding or for non-occlusive PVT, supportive care, and serial ultrasonography monitoring may be appropriate. An expert panel recommends annual screening for evolving portal hypertension for ≥5 years post-diagnosis (38).

Anticoagulation is not indicated for cavernous transformation due to the high rate of variceal bleeding. These children should be referred to gastroenterology for measures to prevent variceal bleeding, which may include: (a) β-adrenergic antagonists, (b) endoscopic variceal band ligation, or (c) endoscopic sclerotherapy (52). Creation of a vascular shunt between the mesenteric vein and left portal vein (Rex shunt) can reduce portal hypertension and restore portal venous flow through the liver (52). Rex shunts are associated with improved growth and cognitive performance, decreased hypersplenism, and prevention of hepatopulmonary syndrome (53–55). Rex shunts are thus recommended for both primary and secondary prophylaxis of variceal bleeding in children with portal hypertension (56).

## Mesenteric Vein Thrombosis (MVT)

Mesenteric vein thrombosis (Figure 1) is exceedingly rare in children with literature limited to case reports and series (57–60). Pancreatitis, surgery, trauma, and oral contraceptives have been associated with pediatric MVT. Early detection and aggressive management are imperative to prevent thrombus progression, bowel infarction, and death (58).

Symptoms of acute MVT are dependent on thrombus size and location, as well as depth of bowel-wall ischemia and include abdominal pain, tenderness, nausea, vomiting, diarrhea, and hematochezia (61). Lactic acidosis may be seen late in the disease course, after bowel infarction has ensued. Early diagnosis is dependent on appropriate imaging studies in symptomatic patients. Plain films are typically non-specific but may include dilated, thickened bowel loops, and multiple air-fluid levels suggestive of ileus. Pneumatosis intestinalis, portal vein gas, and free peritoneal air may be seen later and are characteristic of bowel infarction (61). Doppler ultrasonography may be attempted, but it is often insufficient to adequately evaluate the mesenteric vein. Thus, CT with contrast is preferred and able to confirm the diagnosis in ~90% of cases (62). CT may simultaneously evaluate for bowel infarction and extent.

The rarity of pediatric MVT impairs the development of evidence-based treatment guidelines. In general, management includes exploratory laparotomy with resection of necrotic bowel followed by post-operative anticoagulation. Multiple adult studies have demonstrated that anticoagulation is effective in recanalization of acute MVT and in preventing thrombus progression and recurrence (63–65). Thus, consensus guidelines endorse early anticoagulation for acute MVT in adults; but do not specify duration of therapy (66). In children, we suggest 3 months of anticoagulation for clearly provoked clots, and 6–12 months of therapy for unprovoked clots (or if the initial risk factor is unresolved). There are insufficient data to support thrombolytic therapy for pediatric MVT.

## Budd–Chiari Syndrome (BCS) (Hepatic Vein Thrombosis)

Budd–Chiari syndrome is most commonly diagnosed in adults and results from hepatic venous outflow tract obstruction (50, 67). In adults, the obstruction may occur secondary to extrinsic compression of the hepatic veins or hepatic segment of the IVC (segment 5; Figure 1), most commonly by the mass effect of hepatocellular carcinoma, whereas primary thrombosis is often secondary to JAK2 mutated myeloproliferative disease (~50% of cases) (67). In children, obstruction of the hepatic veins with or without obstruction of IVC segment 5 has been described. The obstruction may occur due to a membrane occluding the vascular lumen, the origins of which are debated (50, 68). An autopsy study of the histopathology of this lesion in adults suggests that the membrane is composed of a fibrous laminar structure derived from organized subacute and chronic thrombi, suggesting thrombotic disease as the underlying etiology (69). This impression is consistent with observations that severe thrombophilias (e.g., antithrombin deficiency, protein C deficiency, JAK2 mutations) are more common than expected in both adult and pediatric series (Table 1) (50, 51, 70). In the case of membranous obstruction, thrombosis is thought to evolve from venostasis; whereas in the case of primary IVC thrombosis, hepatic vein thrombosis may arise secondary to direct extension of thrombus into the intrahepatic vessels. Other causes of hepatic venous obstruction, including sinusoidal obstruction syndrome, should be excluded. BCS is uniformly fatal without treatment (67).

In both pediatric and adult BCS, there is a male predominance, which in pediatrics is ~1.8 male:female (51). Most cases present with subacute or chronic symptoms that include insidious onset of abdominal distention (ascites), abdominal wall collateral veins, and portal hypertension (50, 51, 68). Less common symptoms include GI bleeding, lower extremity edema, jaundice, hepatosplenomegaly, or liver failure (in the acute or fulminant form of the disease) (50, 51, 68). Pediatric BCS has been reported as a complication of liver transplantation, ventriculoatrial shunt placement, and major abdominal surgery as well as medical conditions such as nephrotic syndrome (NS), ulcerative colitis, and liver abscess (50, 71–75).

Doppler ultrasonography has a diagnostic sensitivity of over 85% and is considered to be the frontline imaging modality for BCS (76, 77). CT and MRI scans are usually reserved for inconclusive cases. Rarely, when a diagnosis cannot be established by non-invasive imaging modalities, the patient may need venography and/or liver biopsy (78). There are no well-designed trials of anticoagulant therapy (without procedural intervention) for BCS in adults or children (50, 67). In adults, there is retrospective data suggesting a beneficial effect, but this is likely restricted to patients with less severe disease (67, 79, 80). Meanwhile, in children, the anecdotal experience has been disappointing (50). Interventional modalities are thus favored, with several pediatric series reporting promising results (50, 51, 81, 82). Potential interventions include balloon venoplasty, stent placement, mesocaval shunt, portocaval shunt, pericardial patch reconstruction, and transjugular intrahepatic portosystemic shunt (TIPS). The favored approach is dependent upon the extent of pathology; with venoplasty/stent placement favored for patients with short-segment obstructions whereas TIPS is favored in those with long-segment obstructions (51). Regardless of the approach, long-term anticoagulant or antiplatelet therapy is indicated post-procedure to maintain patency. For patients presenting with fulminant hepatic failure or failing intervention, liver transplantation may be lifesaving. Although there are no outcomes data from well-designed pediatric trials of these interventions, in adults their use has resulted in improved survival to 80% at 5 years (67).

## Renal Vein Thrombosis (RVT)

Renal vein thrombosis (Figure 1) is principally a neonatal disease accounting for 15–20% of neonatal VTE (46, 83, 84). The incidence of neonatal RVT is ~2.2/100,000 live births, and 0.5/1,000 NICU admissions (84, 85). Low renal perfusion pressure, natural anticoagulant deficiency, and renal venous anatomy predispose the neonatal kidney to thrombosis (86). Neonatal RVT originates in the arcuate and interlobular veins with subsequent extension to involve the main renal veins and/or IVC (87). Maternal risk factors for neonatal RVT include diabetes, hypertension, and polyhydramnios; whereas patient risk factors include perinatal asphyxia, hypotension, sepsis, congenital heart disease, and thrombophilia. Neonatal RVT is the most common non-catheter associated VTE in neonates, though UVCs are reported in 15–17% of cases (46, 48).

In older children, RVT has been associated with NS and renal transplantation. The incidence of VTE in adult NS is ~27% with RVT developing in 31%; the highest incidence of RVT (37%) occurring in those with membranous nephropathy (88). In contrast, VTE occurs in only (3%) of childhood-NS cases of which (10%) are RVT, possibly because membranous nephropathy is rare in children (88, 89). It is thought that NS creates an imbalance between urinary loss and synthetic compensation of hemostasis-related proteins (88). In pediatric renal transplantation, RVT has emerged as a leading cause of graft failure (21%) (90). Young recipient age, previous transplant, and pre-transplant peritoneal dialysis are predisposing risk factors.

Most cases of neonatal RVT (67%) are diagnosed within the first 3 days of life (46, 49, 91). Cardinal signs include macroscopic hematuria, thrombocytopenia, and palpable abdominal mass, though the complete triad is seen in only 13–22% of cases (49, 91, 92). Over 50% of cases are associated with varying degrees of renal dysfunction (48). Neonatal RVT is more common in males (67%) and most often unilateral (70%) [left predominant (64%)] (91). IVC extension occurs in 44 and 15% have ipsilateral adrenal hemorrhage.

Neonatal RVT diagnosis requires a high index of suspicion and appropriate imaging. Doppler ultrasonography is the modality of choice and progression of imaging findings have been described (86, 93). Initially, the affected kidney is enlarged with loss of cortico-medullary differentiation and perivascular echogenic streaking thought to be representative of thrombus within the arcuate and interlobular veins. Subsequently, thrombosis may be seen in the renal vein and IVC. Eventually, a significant proportion of kidneys become atrophic. Initial kidney dimensions may be predictive of outcome (49). Each 1 mm increase in kidney length predicts a 3 mL/min/1.73m^2^ loss in glomerular filtration rate; such that kidneys > 6 cm at presentation are rarely salvageable.

Multiple studies have noted a high prevalence of thrombophilic traits (43–68%) in neonates with RVT, particularly FV Leiden (Table 1) (9, 46–49). Neonatal RVT is associated with significant morbidity (91). At 3.7 years, 71% develop renal atrophy, 19% develop hypertension, and 3% require renal replacement therapy. The role of anticoagulation in preventing these complications remains unclear (92). In a study of 23 neonates with RVT, 33% of those receiving heparin developed renal atrophy, compared with 100% in those receiving no anticoagulation. However, subsequent studies revealed no anticoagulation-dependent difference in outcome (85, 91). Current consensus guideline recommendations are: (a) supportive care with serial imaging vs. 6–12 weeks anticoagulation for unilateral RVT with no renal dysfunction and no IVC extension, (b) 6–12 weeks anticoagulation for unilateral RVT with extension into the IVC, and (c) thrombolysis followed by anticoagulation vs. anticoagulation alone for bilateral RVT with evidence of renal dysfunction (23). An expert panel has proposed an RVT risk assessment scale and recommends that the patients be reevaluated biannually for ≥5 years to assess renal function (38).

## Conclusion

Intra-abdominal VTE is infrequent in children. When present, however, they are associated with significant morbidity. The incidence of thrombophilia may be high, thus the risks and benefits of thrombophilia testing should be carefully considered with the patient and family. The approach to therapy is guided by the involved veins and may include thrombolysis, surgery, and/or anticoagulation and warrants a multi-disciplinary approach involving pediatric hematology, interventional radiology, and relevant sub-specialties.

## Author Contributions

Both authors reviewed the literature, wrote, and edited the manuscript. RK wrote the first draft of the Portal, Mesenteric, and Renal Vein sections. BK wrote the first draft of the IVC and Budd–Chiari sections.

## Conflict of Interest Statement

BK has received research support from the CSL Behring Foundation, NovoNordisk A/S, and Bayer Healthcare US and also serves on advisory boards for NovoNordisk A/S and Bayer Healthcare US. RK serves on an advisory board for Bayer Healthcare US. The authors have no actual or potential conflict of interest.
